# Supplementary material for: Efficient removal of nanoplastics from industrial wastewater through synergetic electrophoretic deposition and particle-stabilized foam formation
Source: Nat Commun. 2024 Jun 27;15:5437. doi: 10.1038/s41467-024-48142-2 (PMC11211448; doi:10.1038/s41467-024-48142-2)
Supplement: Supplementary file 1 — Supplementary Information [file 41467_2024_48142_MOESM1_ESM.pdf]

Supplementary information

Efficient removal of nanoplastics from industrial wastewater through synergetic electrophoretic deposition and particle-stabilized foam formation

Amna Abdeljaoued[1,2], Beatriz López Ruiz[1,2], Yikalo-Eyob Tecle[1], Marie Langner[1],

Natalie Bonakdar[2], Gudrun Bleyer[2], Patrik Stenner[1], Nicolas Vogel[2]

1) Particle Processing, Process Technology & Engineering, Evonik Operations GmbH, Rodenbacher Chaussee 4, Wolfgang 63457, Germany.

2) Institute of Particle Technology, Friedrich-Alexander-Universität Erlangen-Nürnberg, Cauerstrasse 4, 91058 Erlangen, Germany.

Corresponding author: nicolas.vogel@fau.de

Content

1. *SUPPLEMENTARY NOTES* 2

Supplementary Note 1. Calculation of the mesh electrode surface 2

Supplementary Note 2. Cost calculation of nanoplastic removal from industrial wastewater by ePhoam process with the up-scaled roller (5 L of wastewater of 1 wt.%) 3

Supplementary Note 3. Carbon footprint estimation 5

Supplementary Note 4. Details of model particles synthesis 7

2. SUPPLEMENTARY FIGURES 8

3. SUPPLEMENTARY TABLES 22

SUPPLEMENTARY REFERENCES 28

1. *Supplementary Notes*

Supplementary Note 1. Calculation of the mesh electrode surface

The calculation of structural surface area of the type of DSA expanded mesh that we have used, was reported in literature[1]. The expanded mesh is divided into two parts: an independent segment and an intersection segment as shown in Supplementary Figure 10. The number of intersection and independent segment can be calculated using the number of pores in the direction of long way of design (LWD) and short way of design (SWD) as shown in Supplementary Figure 10; (LWD: n, SWD: m). The number of non-overlapping pores on each side is equal to the number of pores in the LWD multiplied by the number of pores in the SWD ($n\ m$). s represents strand width (cm), a corresponds to vertical width; b corresponds to horizontal width; t means thickness (cm), and w is pore width (cm). The surface area of the expanded mesh can be calculated as follows, demonstrated by Suppl. Ref. 1:

$$n\ m\ (8\ s\ w + 8\ t\ w + 2\ a\ b) \tag{1}$$

In our case, this formula yields 133.5 cm^2, for n = 5 cm^{-1}; m = 15 cm^{-1}; s = 0.2 cm; w = 0.5 cm; t = 0.2 cm; a = 0.3 cm; b = 0.3 cm.

Supplementary Note 2. Cost calculation of nanoplastic removal from industrial wastewater by ePhoam process with the up-scaled roller (5 L of wastewater of 1 wt.%)

- The power consumption for the process is calculated by multiplying the voltage and the current.

$$20\ \mathrm{V} * 20\ \mathrm{A} = 400\ \mathrm{W} = 0.4\ \mathrm{kW} \tag{2}$$

- The process cleans 5 kg of wastewater (ww) in 2 h which leads to a starting mass flow of 2.5 kg h^{-1}

$$\frac{5\ \mathrm{kg_{ww}}}{2\ \mathrm{h}} = 2.5\ \mathrm{kg_{ww}}\ \mathrm{h^{-1}} \tag{3}$$

- By dividing the power consumption by the start mass flow, the energy consumption per mass flow or per kg of wastewater is calculated.

$$\frac{0.4\ \mathrm{kW}}{2.5\ \mathrm{kg_{ww}}\ \mathrm{h^{-1}}} = 0.16\ \mathrm{kWh}\ \mathrm{kg_{ww}^{-1}} \tag{4}$$

- The current cost of energy (July 2023) is 0.12 € kWh^{-1}, so the cost of the energy consumption for the process is 19.2 € t_{ww}^{-1}

$$0.16\ \mathrm{kWh}\ \mathrm{kg_{ww}^{-1}} * 0.12\ €\ \mathrm{kWh^{-1}} = 0.0192\ €\ \mathrm{kg_{ww}^{-1}} = 19.2\ €\ \mathrm{t_{ww}^{-1}} \tag{5}$$

- The operational expenses (opex) for drying, pumps, cooling, etc. are estimated with 10% of the energy costs according to experience.

$$19.2\ €\ \mathrm{t_{ww}^{-1}} * 0.1 = 1.92\ €\ \mathrm{t_{ww}^{-1}} \tag{6}$$

- For the capital expenditures (capex) of a 10 t_{ww} h^{-1} plant a mass flow of 80,000 t $year^{-1}$ is calculated. A year is estimated by 8,000 h.

$$10\ \mathrm{t_{ww}}\ \mathrm{h^{-1}} * 8{,}000\ \mathrm{h\ year^{-1}} = 80{,}000\ \mathrm{t_{ww}}\ \mathrm{year^{-1}} \tag{7}$$

- The estimated invest for a 10 t_{ww} h^{-1} plant is 3,000,000 € based on experience with a small package unit. With 10 years depreciation time 300,000 € $year^{-1}$ are calculated.

$$\frac{3{,}000{,}000\ €}{10\ \mathrm{years}} = 300{,}000\ €\ \mathrm{year^{-1}} \tag{8}$$

- Per metric ton of wastewater, the capex costs calculate to 3.75 € t_{ww}^{-1}.

$$\frac{300{,}000\ €\ \mathrm{year^{-1}}}{80{,}000\ \mathrm{t_{ww}}\ \mathrm{year^{-1}}} = 3.75\ €\ \mathrm{t_{ww}^{-1}} \tag{9}$$

- The recovered mass of polymer for the 10 t h^{-1} plant can be calculated by multiplying the mass flow with the starting concentration of polymer in the wastewater which is 1 wt.% and the removal rate of 91%.

$$10\ \mathrm{t_{ww}\ h^{-1}} * 0.01\ \mathrm{t_{polymer}\ t_{ww}^{-1}} *\ 0.91 = 0.091\ \mathrm{t_{polymer}\ h^{-1}} \quad (10)$$

$$\frac{0.091\ \mathrm{t_{polymer}\ h^{-1}}}{10\ \mathrm{t_{ww}\ h^{-1}}} = 0.0091\ \mathrm{t_{polymer}\ t_{ww}^{-1}} \quad (11)$$

- With a payback for the polymer of 400 € $t_{polymer}^{-1}$,a payback of 3.64 € t_{ww}^{-1} is generated.

$$0.0091\ \mathrm{t_{polymer}\ t_{ww}^{-1}} * 400\ € \ \mathrm{t_{polymer}^{-1}} = 3.64\ € \ \mathrm{t_{ww}^{-1}} \quad (12)$$

- Combined the costs for energy consumption, capex and opex with the payback for the polymer sum up to 21.23 € t_{ww}^{-1}.

$$19.2\ € \ \mathrm{t_{ww}^{-1}} + 1.92\ € \ \mathrm{t_{ww}^{-1}} + 3.75\ € \ \mathrm{t_{ww}^{-1}} - 3.64\ € \ \mathrm{t_{ww}^{-1}} = 21.23\ € \ \mathrm{t_{ww}^{-1}} \quad (13)$$

- Compared to the alternative process that is used now (incineration of wastewater) and costs 200 € t_{ww}^{-1} 178.77 € per ton of wastewater are saved.

$$200\ € \ \mathrm{t_{ww}^{-1}} - 21.23\ € \ \mathrm{t_{ww}^{-1}} = 178.77\ € \ \mathrm{t_{ww}^{-1}} \quad (14)$$

Supplementary Note 3. Carbon footprint estimation

We aim to compare the introduced ePhoam process with the current treatment of wastewater, which uses the incineration process. For this carbon footprint estimation of wastewater treatment, we consider direct and indirect carbon emissions caused by electricity production off site. As there is little difference in inputs and by-products, their contributions were considered neglectable. The calculation was performed for a production site in Germany in 2023. The emissions are calculated per m^3 wastewater. The data and assumption are based on process and energy data provided by Evonik in August 2023. Emission factors were obtained from EU Regulation 601/2012 on the monitoring and reporting of GHG emissions[2]

❖ Using ePhoam process through an up-scaled roller setup (5 L, 2 h)

- The roller treats $5 * 10^{-3}\ \mathrm{m^3}$ of wastewater (ww) for 2 h.

$$\frac{2\ \mathrm{h}}{5 * 10^{-3}\ \mathrm{m^3_{ww}}} = 400\ \mathrm{h\ m^{-3}_{ww}} \tag{15}$$

- The rollers power demand is calculated by multiplying current and voltage.

$$20\ \mathrm{V} * 20\ \mathrm{A} = 400\ \mathrm{W} = 0.4\ \mathrm{kW} \tag{16}$$

- By multiplying the power demand with the time per m³ wastewater the energy needed for one cubic meter of wastewater is calculated.

$$0.4\ \mathrm{kW} * 400\ \mathrm{h\ m^{-3}_{ww}} = 160\ \mathrm{kWh\ m^{-3}_{ww}} \tag{17}$$

- The motor that turns the roller has a power demand of 0.18 kW. That multiplied by the time the process needs for cubic meter of wastewater calculates the energy used by the motor per cubic meter wastewater.

$$0.18\ \mathrm{kW} * 400\ \mathrm{h\ m^{-3}_{ww}} = 72\ \mathrm{kWh\ m^{-3}_{ww}} \tag{18}$$

- The sum of the energy used by the process is $232\ \mathrm{kWh\ m^{-3}_{ww}}$

$$72\ \mathrm{kWh\ m^{-3}_{ww}} + 160\ \mathrm{kWh\ m^{-3}_{ww}} = 232\ \mathrm{kWh\ m^{-3}_{ww}} \tag{19}$$

- The carbon footprint of the process is calculated by multiplying the total energy demand by the CO_2 emissions per kWh for Germany which is 0.58 $*10^{-3}\ \mathrm{t_{CO_2} kWh^{-1}}$

$$232\ \mathrm{kWh\ m^{-3}_{ww}} * 0.58 * 10^{-3}\ \mathrm{t_{CO_2} kWh^{-1}} = 0.13456\ \mathrm{t_{CO_2}\ m^{-3}_{ww}} \tag{20}$$

❖ Using the incineration process

- For incinerating one m³ wastewater with a low particle content 240 Nm³ (standard condition reference volume) of methane are used.
- With a conversion of $10.55\ \mathrm{kWh\ Nm^{-3}_{methane}}$ that equal $5.497\ \mathrm{MWh\ m^{-3}_{ww}}$

$$240\ \mathrm{Nm^{3}_{methane}\ m^{-3}_{ww}} * 10.55\ \mathrm{kWh\ Nm^{-3}_{methane}} = 2532\ \mathrm{kWh\ m^{-3}_{ww}} = 2.532\ \mathrm{MWh\ m^{-3}_{ww}} \tag{21}$$

- The incineration of the plastic itself produces $0.2\ t_{CO_2\,e}\ MWh^{-1}$

$$0.2\ t_{CO_2e}\,MWh^{-1} *\ 2.532\ MWh\ m_{ww}^{-3}\ = 0.5064\ t_{CO_2e}\,m_{ww}^{-3} \tag{22}$$

- For the raw material of the natural gas the emission factor is $0.46\ t_{CO_2}t_{methane}^{-1}$ and the density of methane is $0.718\ kg\ m^{-3} = 0.718 *\ 10^{-3}\ t\ m^{-3}$

$$0.46\ t_{CO_2}t_{methane}^{-1} *\ 240\ \ Nm_{methane}^{3}\ m_{ww}^{-3} * 0.718 *\ 10^{-3}t_{methane}Nm_{methane}^{-3}\ = 0.0793\ t_{CO_2}m_{ww}^{-3} \tag{23}$$

- This sums up to a carbon footprint of $0.5857\ t_{CO_2e}\,m_{ww}^{-3}$

$$(0.5064\ + 0.0793)\ t_{CO_2e}\,m_{ww}^{-3}\ = 0.5857\ \ t_{CO_2e}\,m_{ww}^{-3} \tag{24}$$

Compared to the ePhoam process, $0.45114\ \ t_{CO_2e}\,t_{ww}^{-1}$ would be saved.

$$(0.5857\ - 0.13456)\ t_{CO_2e}\,m_{ww}^{-3} = 0.45114\ t_{CO_2e}\,t_{ww}^{-1} \tag{25}$$

Using the ePhoam process, we estimate a carbon footprint reduction by almost 77%, without considering the reuse of the recovered particles.

Supplementary Note 4. Details of model particles synthesis

PS-acrylic acid synthesis

Briefly, 1000 mL of water was heated to a temperature of 75 °C in a three-necked-flask equipped with a reflux condenser. During the heating time and the reaction, the system was constantly flushed with nitrogen under stirring at 500 rpm. When the reaction temperature was reached, 30 g of styrene was added. After 10 min, 0.40 g of acrylic acid and 0.40 g of ammonium persulfate, both dissolved in 5 mL of water, were added in 10 min intervals. After 22 h, the nitrogen flow and heating were stopped, and the colloid was left to cool.

PS-amidine synthesis

In a three-necked-flask equipped with a reflux condenser 250 mL of water was heated to 60 °C. During the heating time and the reaction, the system was constantly flushed with nitrogen under stirring at 675 rpm. 20 g of styrene was added when the reaction temperature was reached. After 5 min, 0.63 g of trimethyl-ammonium chloride (vinylbenzyl) and 0.28 g of 2,2-azobis (2-methylpropionamidine) dihydrochloride, both dissolved in 5 mL of water, were added in 5 min intervals. The nitrogen flow and heating were stopped after 22 h and the colloid was left to cool.

PS sodium 4-vinylbenzenesulfonate synthesis

250 mL of water was heated to a temperature of 80 °C in a three-necked-flask equipped with a reflux condenser. During the heating time and the reaction, the system was constantly flushed with nitrogen. When the reaction temperature was reached, 10 g of styrene was added. After 10 min, 0.052 g of sodium 4-vinylbenzenesulfonate and 0.1 g of ammonium persulfate, both dissolved in 5 mL of water, were added in 5 min intervals. After 22 h, the nitrogen flow and heating were stopped, and the colloid was left to cool.

PBMA-acrylic acid synthesis

240 mL of water was heated to a temperature of 80° C in a three-necked-flask equipped with a reflux condenser. During the heating time and the reaction, the system was constantly flushed with nitrogen under stirring at 700 rpm. 11 g of butyl methacrylate was added, when the reaction temperature was reached. After 10 min, 0.63 g of acrylic acid and 1.3 g of ammonium persulfate, both dissolved in 5 mL of water, were added in 5 min intervals. After 22 h, the nitrogen flow and heating were stopped, and the colloid was left to cool.

2. Supplementary Figures

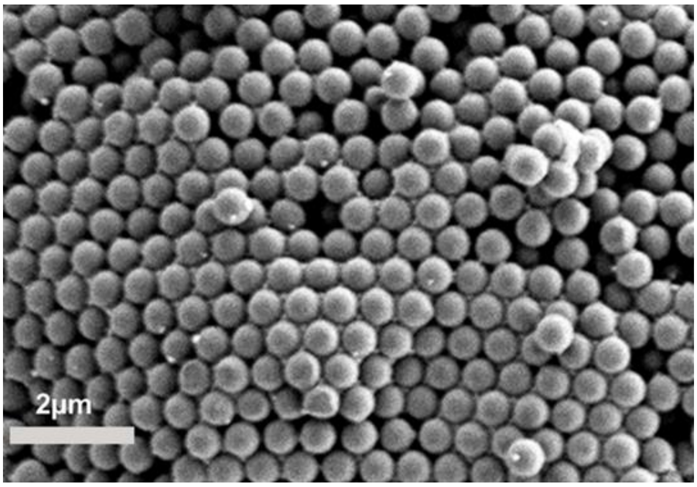

Supplementary Figure 1: Scanning electron microscopy (SEM) image of the PMMA particles of the model system at 10K magnification.

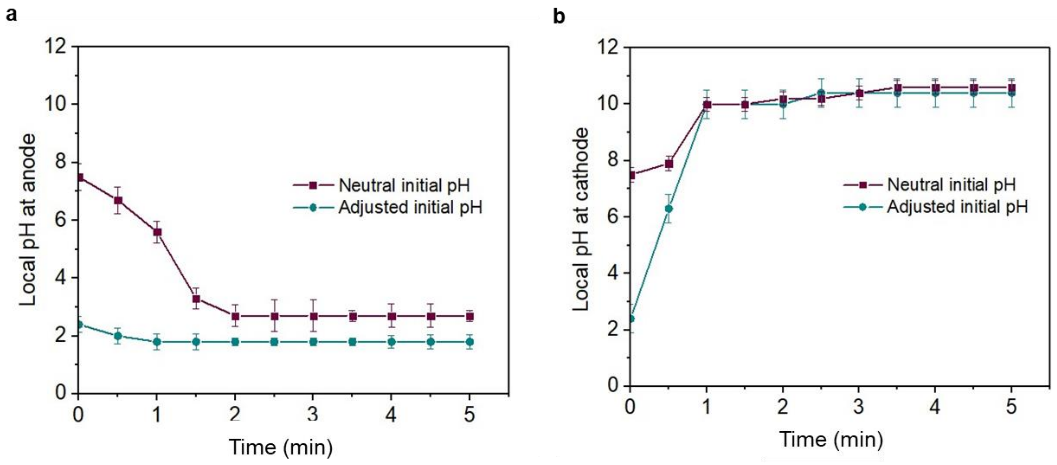

Supplementary Figure 2: Local pH shift during Water electrolysis, **a** Local pH in the vicinity of the anode **b** Local pH in the vicinity of the cathode; for a neutral bulk pH and a pH adjusted the match the experimental conditions in the presence of the model colloidal particles. Error bars represent standard deviation, *n* = 3 independent replicates.

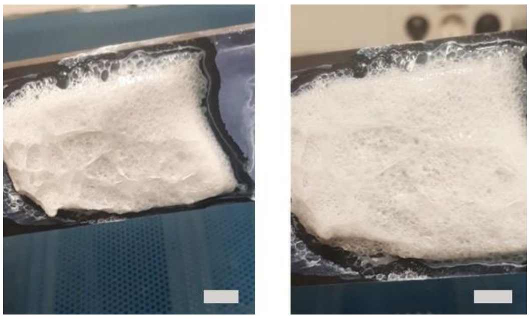

Supplementary Figure 3: Photographs of wet stable foam on the anode surface. Scale bars= 1 cm.

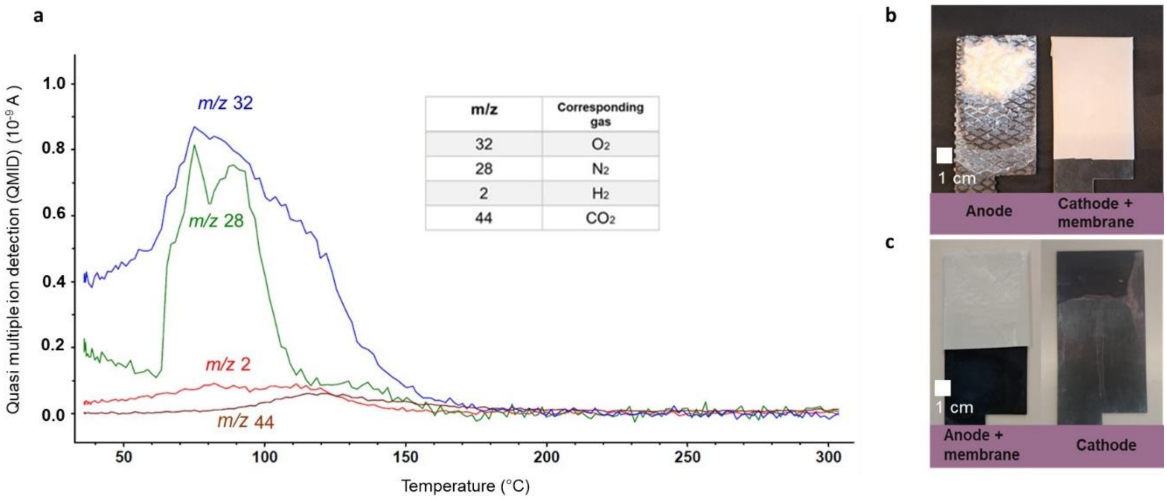

Supplementary Figure 4: **a** Individual mass traces plotted over temperature based on EGA analysis. **b, c** Photos of both electrodes after the separation process using a nylon membrane (0.1 µm). Blue: O_2; Green: N_2; red:H_2 and brown: CO_2. **b** firstly covering the cathode and **c** secondly covering the anode. The membrane blocks access of the colloidal particles to the electrode surface and thus to the local environment of the pH change. In addition, gas evolution mainly occurred at the electrode/membrane interface, and thus in absence of the colloidal particles. When the anode is uncovered, large amounts of foam can be seen **(b)**. When the anode is covered, however, no foam appears, neither at the anode, nor at the cathode **(c)**.

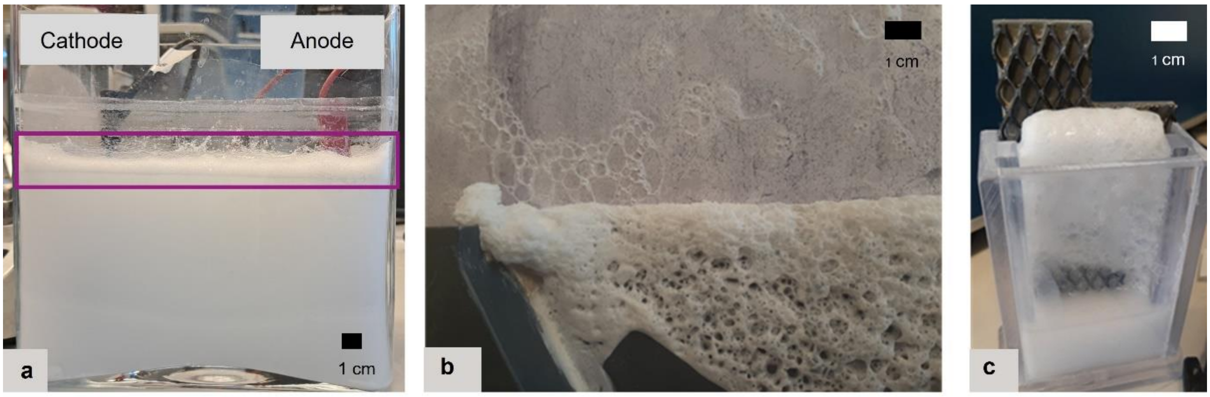

Supplementary Figure 5: Comparison to conventional electroflotation. **a** Setup for electroflotation experiment using the PMMA model system and the mixed metal oxides electrodes (DSA). Note the limited amount of observed foam, indicating instable foam formation. The pH in the boxed area was similar to that of the bulk solution (pH ≈ 2.4). **b** ePhoam process; Stable foam on the anode surface in the roller setup being collected in the doctor blade part. **c** Stable foam formed on the anode surface in the discontinuous setup. Scale bars = 1 cm.

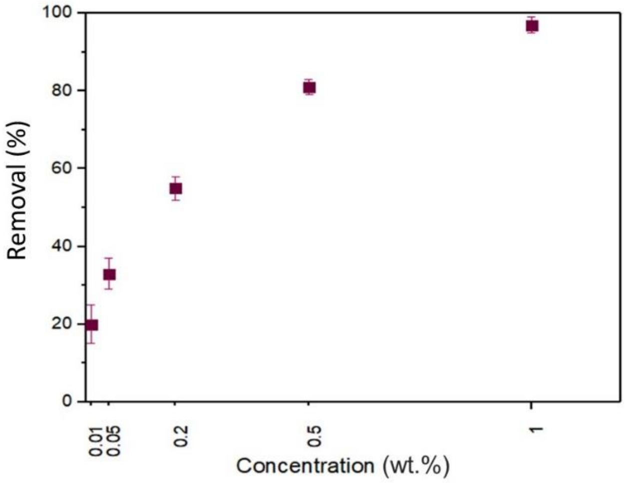

Supplementary Figure 6: Impact of the concentration on the removal efficiency. The separation efficiency was proportional to the concentration when all other parameters are fixed. Note that the parameters were optimized for efficient removal at high concentrations as occurring in industrial wastewater. Optimization of the parameters, such as increased time and stirring of the dispersion can significantly improve removal at lower concentrations. Error bars represent standard deviation, n = 3 independent replicates.

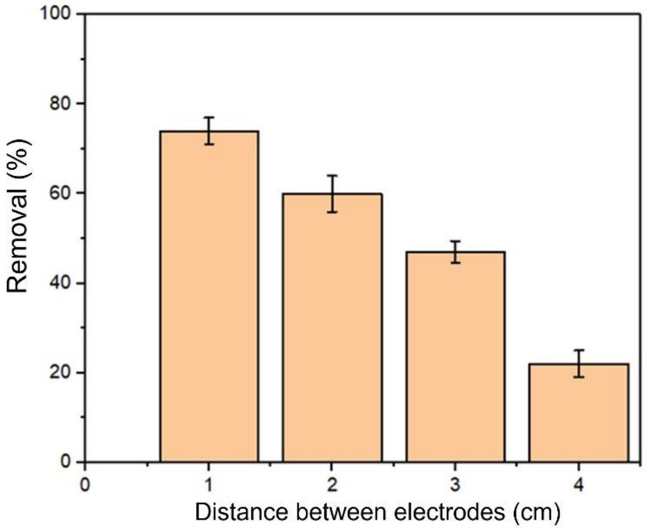

Supplementary Figure 7: Impact of the distance between electrodes. The removal efficiency, at an applied 40 V, was inversely proportional to the interelectrode distance, which in our setup we fixed to 1 cm as compromise between efficient separation and the ability to remove particle-stabilized foam. Error bars represent standard deviation, *n* = 3 independent replicates.

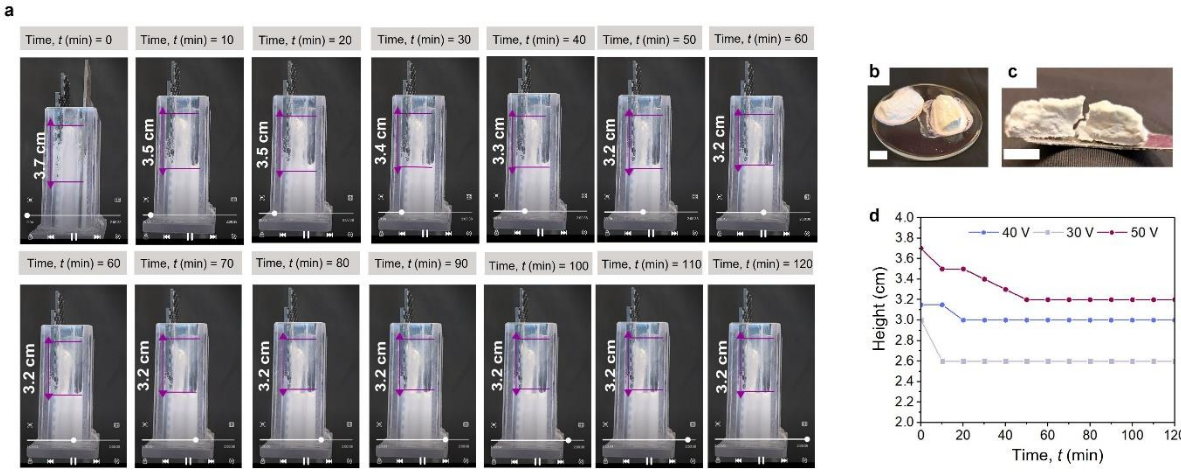

Supplementary Figure 8: **a** Photos of the stable foam standing on the anode surface inside the electrolytic cell filmed for 2 h and captured as photos every 10 min at different voltages (*V*= 50 V in this figure). The produced foam is highlighted with arrows. **b** Foam samples at room temperature after 24 hours, and **c** dried stable foam after one week. Scale bars = 1 cm. **d** Particle stabilized foam stability assessment through the foam height versus time at different voltages. Dark pink: 50 V; blue: 40 V and light purple: 30 V.

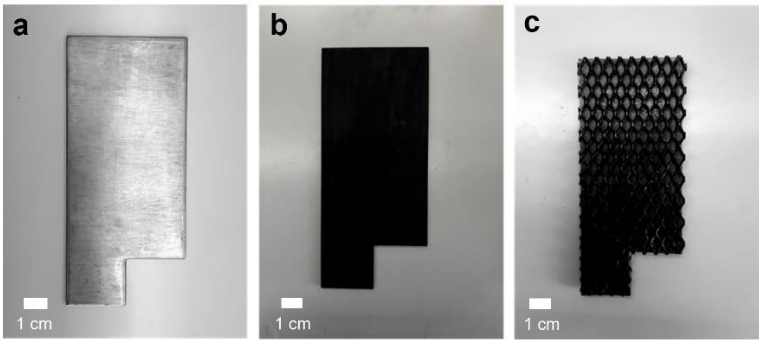

Supplementary Figure 9: Different electrode types used in the separation process. **a** Flat stainless steel; **b** Flat Dimensionally stable anode (DSA); **c** Mesh dimensionally stable anode (DSA).

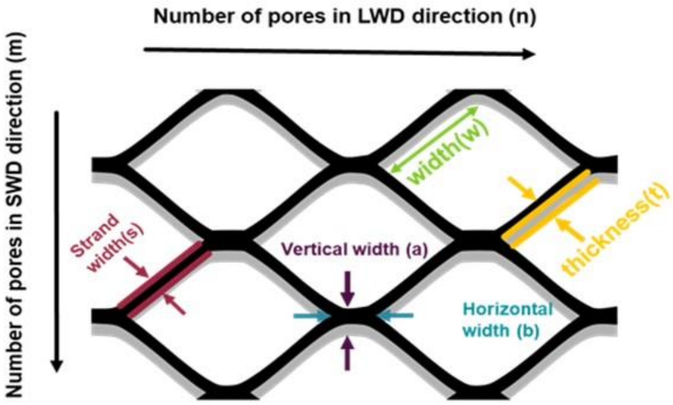

Supplementary Figure 10: Structural surface area of expanded mesh: where *a* and *b* are vertical and horizontal width of rhombus (cm), respectively. The number of intersection and independent segment can be calculated using the number of pores (LWD: *n*, SWD: *m*) in the direction of long way of design (LWD) and short way of design (SWD) as shown in the scheme above. The number of non-overlapping pores on each side is equal to the number of pores in the LWD multiplied by the number of pores in the SWD. *s* and *t* stand for the strand width and thickness respectively, *w* represents the pore width[1].

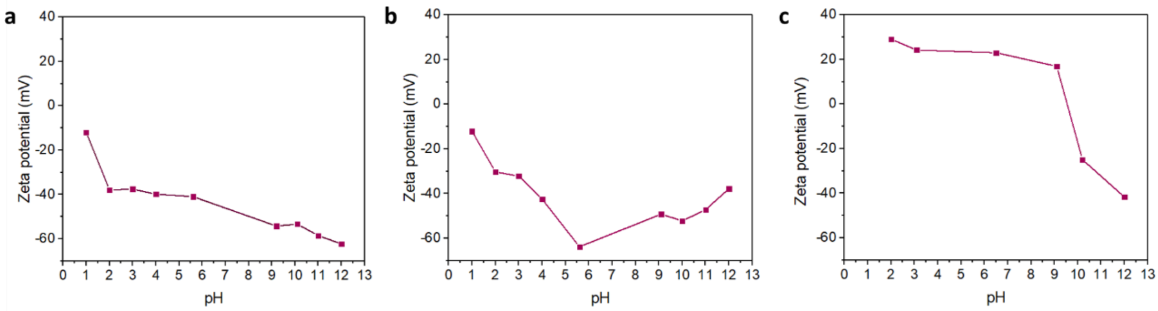

Supplementary Figure 11: Zeta potential of PS nanoplastic model dispersions; **a** PS-carboxylate **b** PS-sulfonate **c** PS-amidine.

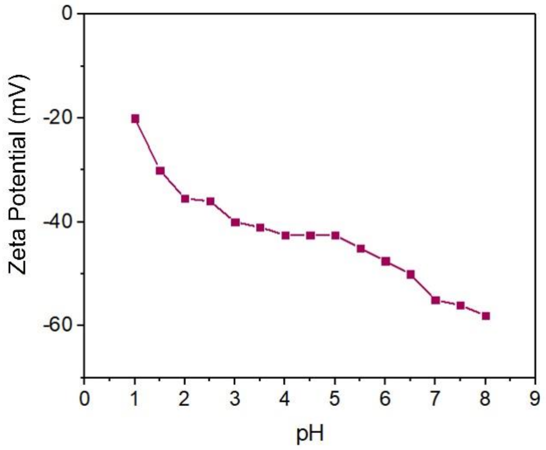

Supplementary Figure 12: Zeta potential as a function of pH for the industrial wastewater containing PMMA particles.

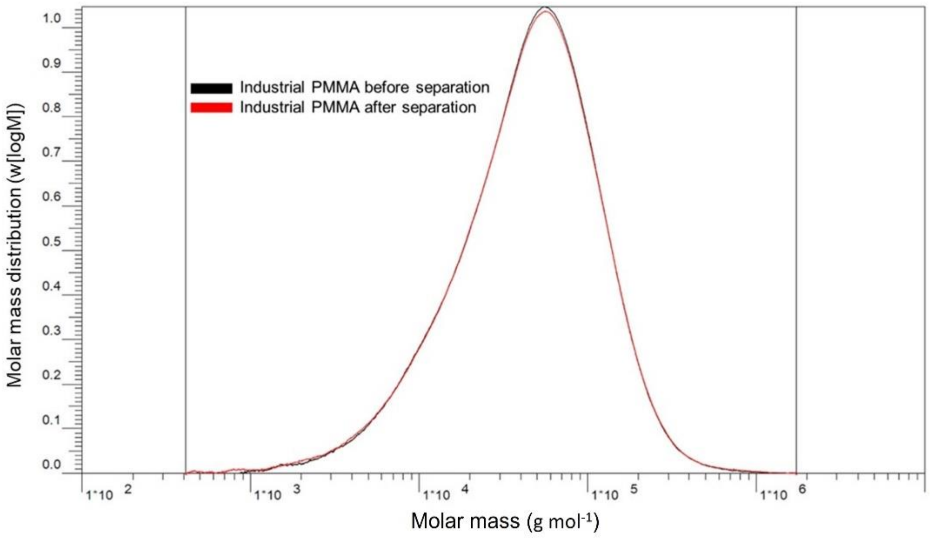

Supplementary Figure 13: Molar mass distribution measured using gel permeation chromatography of PMMA particles recovered from industrial wastewater before and after the separation.

a

b

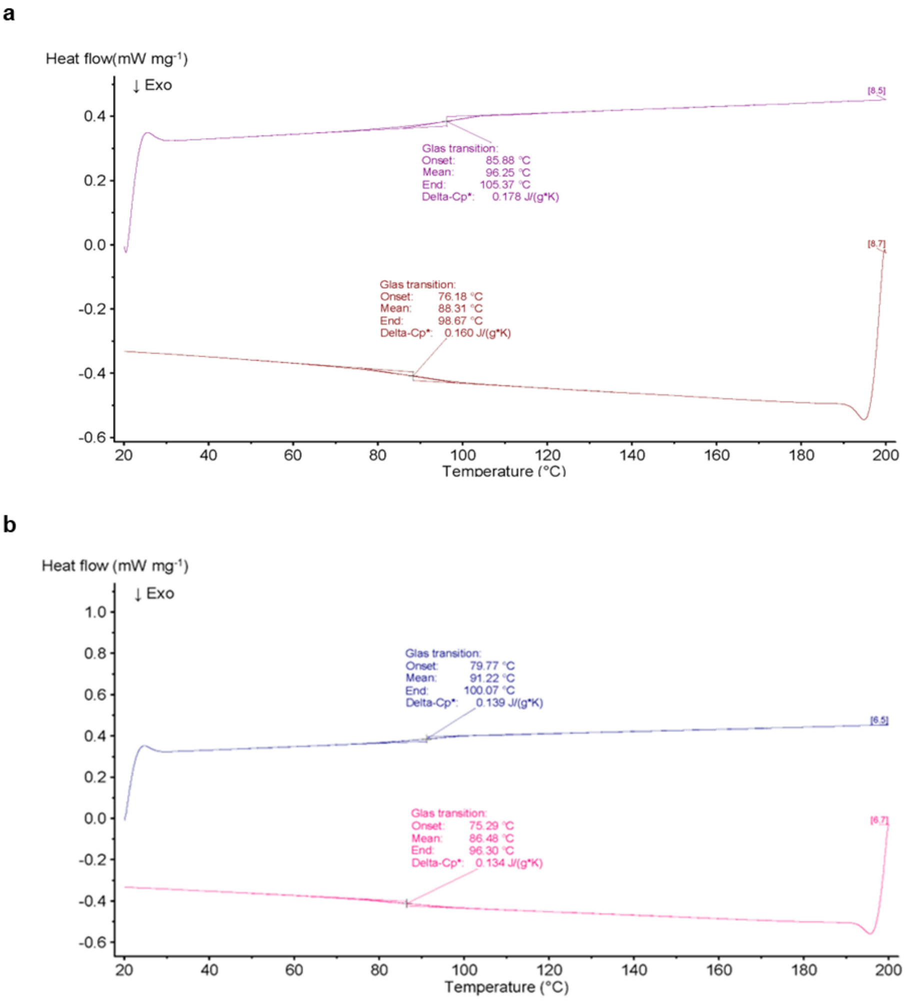

Supplementary Figure 14: Differential scanning calorimetry (DSC) characterization of the PMMA particles of industrial wastewater **a** before and **b** after the separation process.

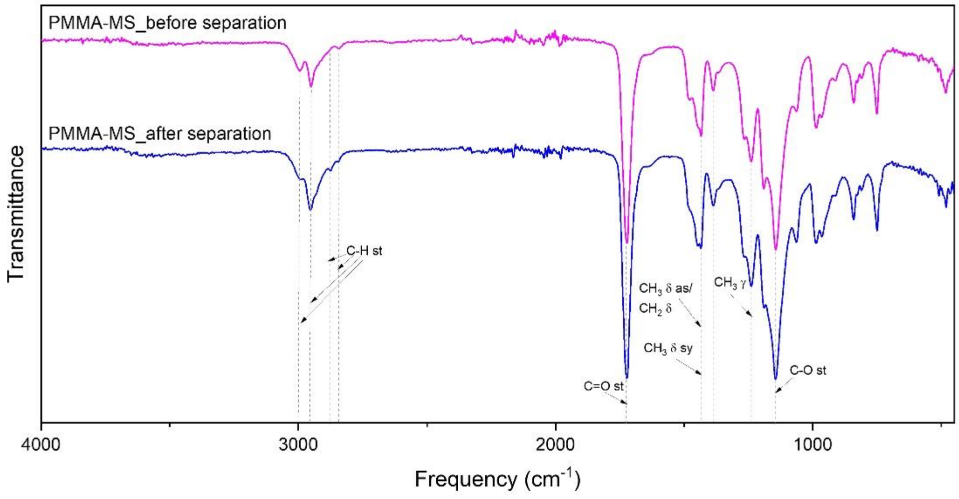

Supplementary Figure 15: Attenuated total reflectance infrared (ATR-IR) spectroscopy analyses of the PMMA particles of industrial wastewater before and after the separation process. St: Stretching vibration; sy: symmetric stretching; as: asymmetric stretching; δ: deformation vibration; γ: skeletal vibration.

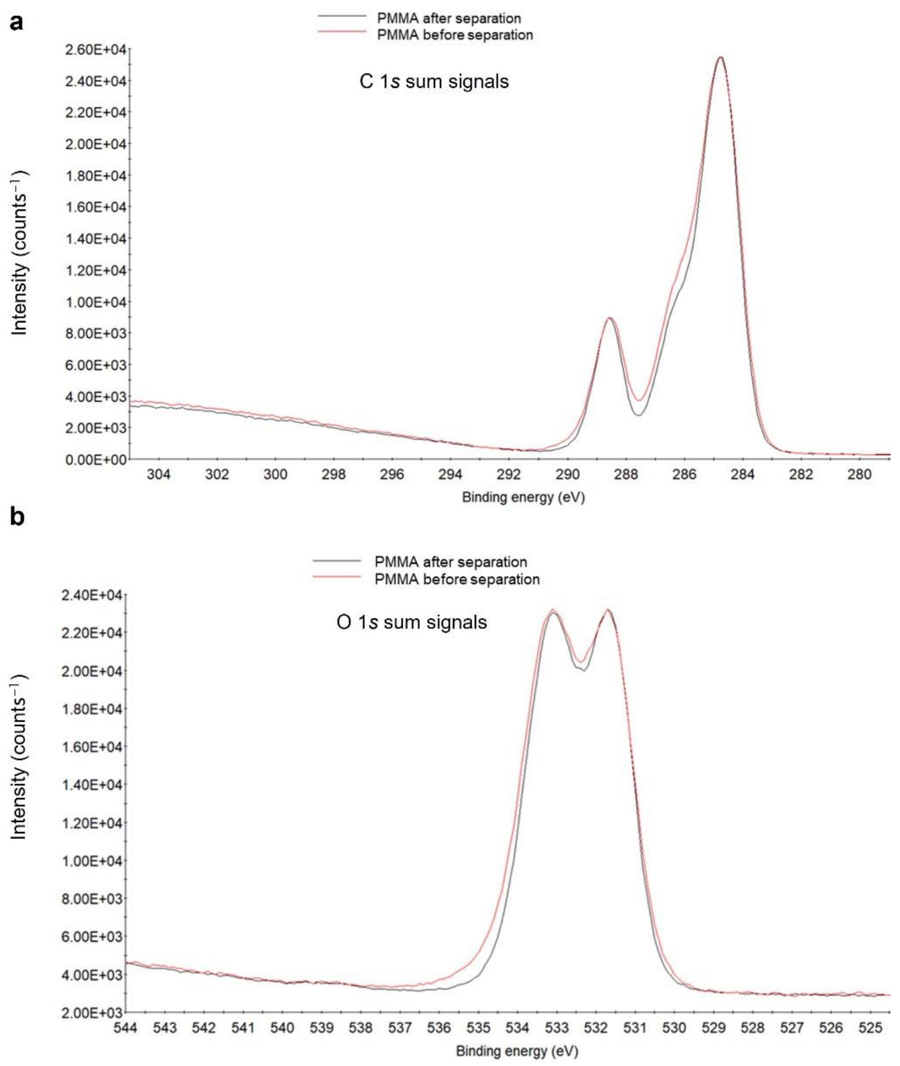

Supplementary Figure 16: X-ray photoelectron spectrometry of PMMA particles of the industrial wastewater before and after the separation process **a** Overlay plot of the C 1*s*- sum signals **b** Overlay plot of the O 1*s*- sum signals.

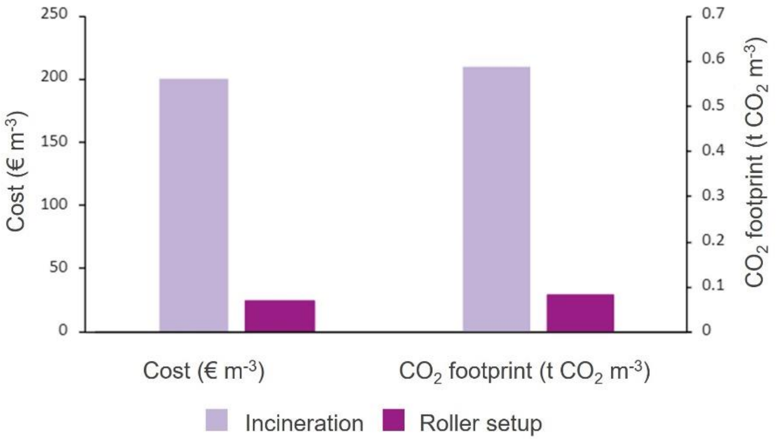

Supplementary Figure 17: Comparison of cost calculation and carbon footprint estimation between the current incineration process and the ePhoam process using the upscaled roller setup shown in Fig. 7 of the main text.

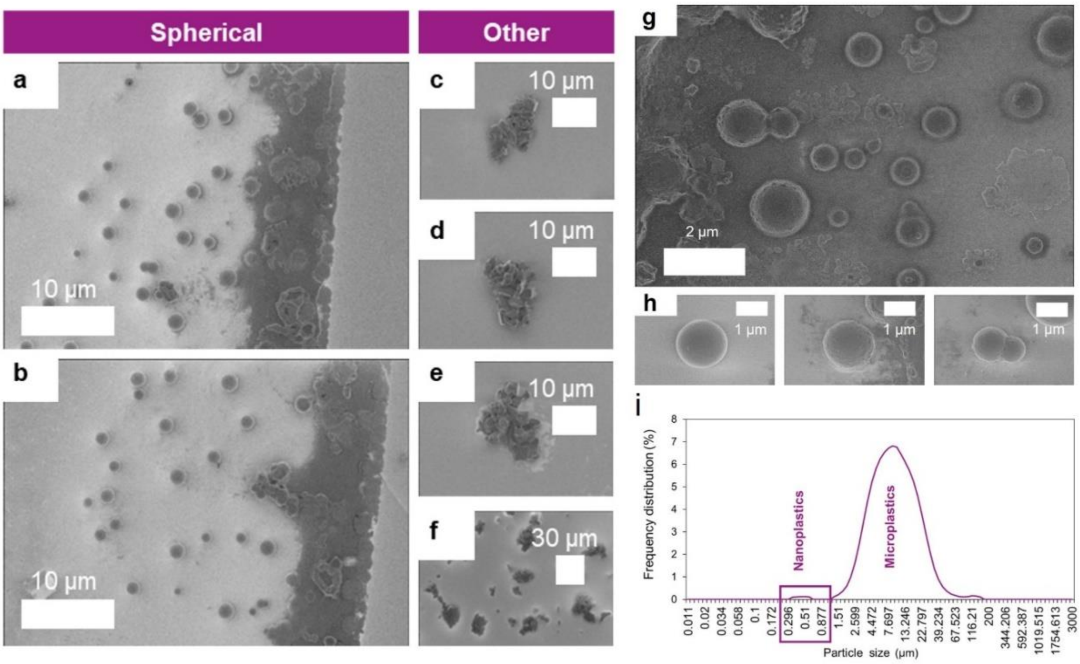

Supplementary Figure 18: Particle size and shape characterization of the wastewater generated in the eye glass and contact lens polishing. **a-f** SEM images of the particles found in the wastewater, showing different shapes and structures; **g,h** SEM images of differently-sized spherical particles. **i** Particle size distribution measured by particle size distribution analyzer (Partica LA-950V2 laser diffraction).

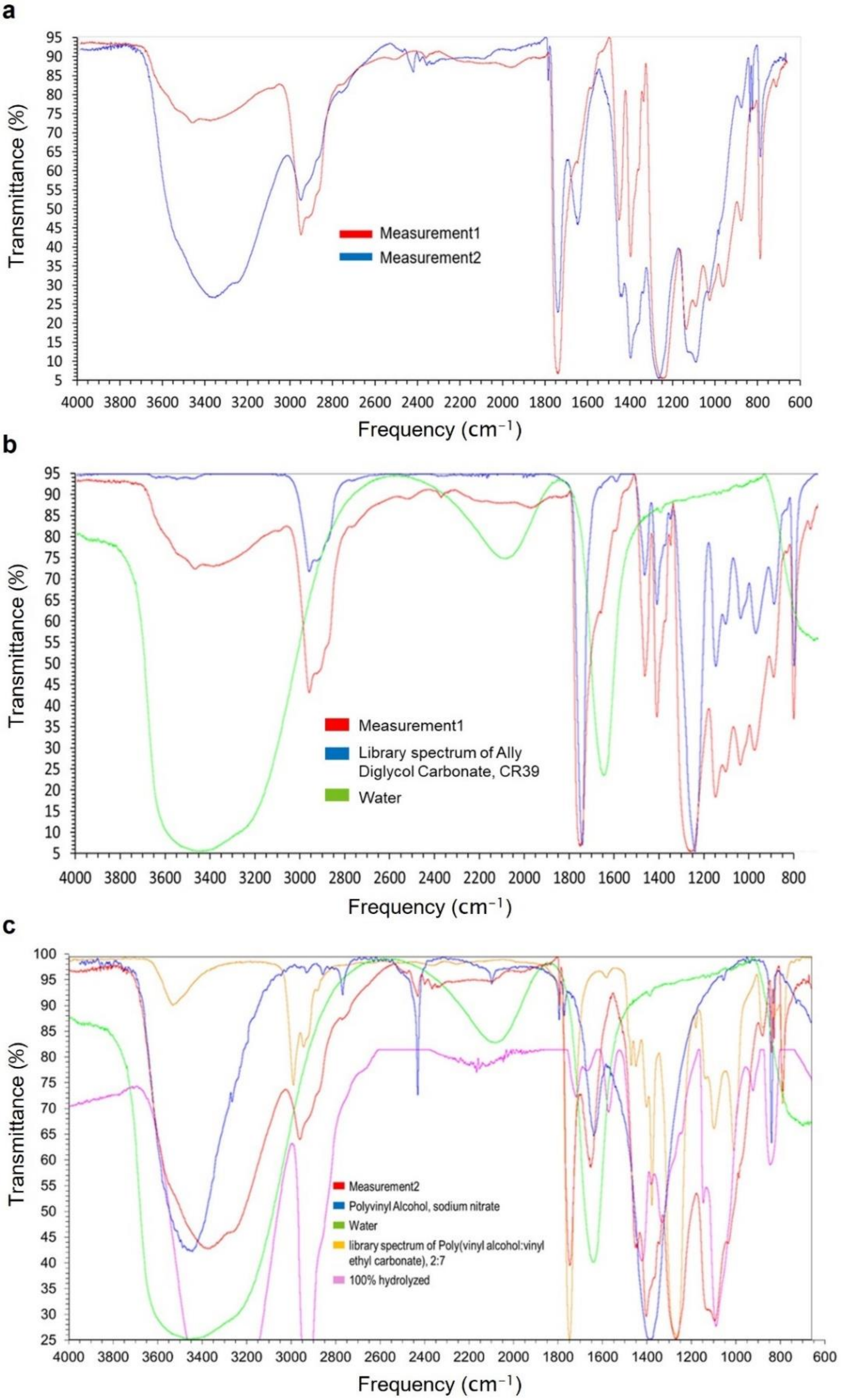
a
Transmittance (%)
Frequency (cm⁻¹)
Measurement1
Measurement2
b
Transmittance (%)
Frequency (cm⁻¹)
Measurement1
Library spectrum of Ally Diglycol Carbonate, CR39
Water
c
Transmittance (%)
Frequency (cm⁻¹)
Measurement2
Polyvinyl Alcohol, sodium nitrate
Water
library spectrum of Poly(vinyl alcohol:vinyl ethyl carbonate), 2:7
100% hydrolyzed

Supplementary Figure 19: Fourier-transform infrared (FTIR) spectroscopy of eyeglass and contact lenses polishing wastewater. **a** IR-spectra taken from 2 different samples of the same wastewater after two separate separation runs; **b** IR-spectrum of measurement 1 and its comparison with a library spectrum of allyl diglycol carbonate, CR39 and water spectrum. **c** IR-spectrum of measurement 2 overlaid with a library spectrum of poly (vinyl alcohol: vinyl ethyl carbonate), 2:7 (yellow), water (green), polyvinyl alcohol, sodium nitrate (blue) and 100% hydrolyzed (pink). Measurement 1 shows relatively good agreement with an available library spectrum of poly allyl diglycol carbonate (PADC) polymer, commercially known as (CR39) **(a; b)**. The three main peaks for the polycarbonate (PC) were also present, (C=O) peak of carbonyl at ≈ 1750 cm^{-1}, the (O-C-O) stretch at ≈ 1230 cm^{-1}, and the (C-O-C) stretch at ≈ 1050 cm^{-1}. The peak at 789 cm^{-1} is assigned to the (C-H) bond. IR peak for water in (PADC) polymer has always been present. CR39 plastic is known by its property for water uptake from environment[3]. The peak at ≈ 3500 cm^{-1} is therefore assigned to the weak hydrogen bond of the water molecule to the polymer molecule[4]. Measurement 2 **(b; c)** shows a more complex spectrum than measurement 1, as the polishing wastewater is very heterogeneous. Organic and inorganic additives and contaminants adsorbed on MPs and NPs were expected to be detected in the analysis. There are absorptions from organic carbonate and in the carbonyl range (≈ 1750 cm^{-1}), absorptions for H_2O at ≈ 3550 cm^{-1}, and absorptions indicative of inorganic nitrate (≈ 1395 cm^{-1}) were also visible.

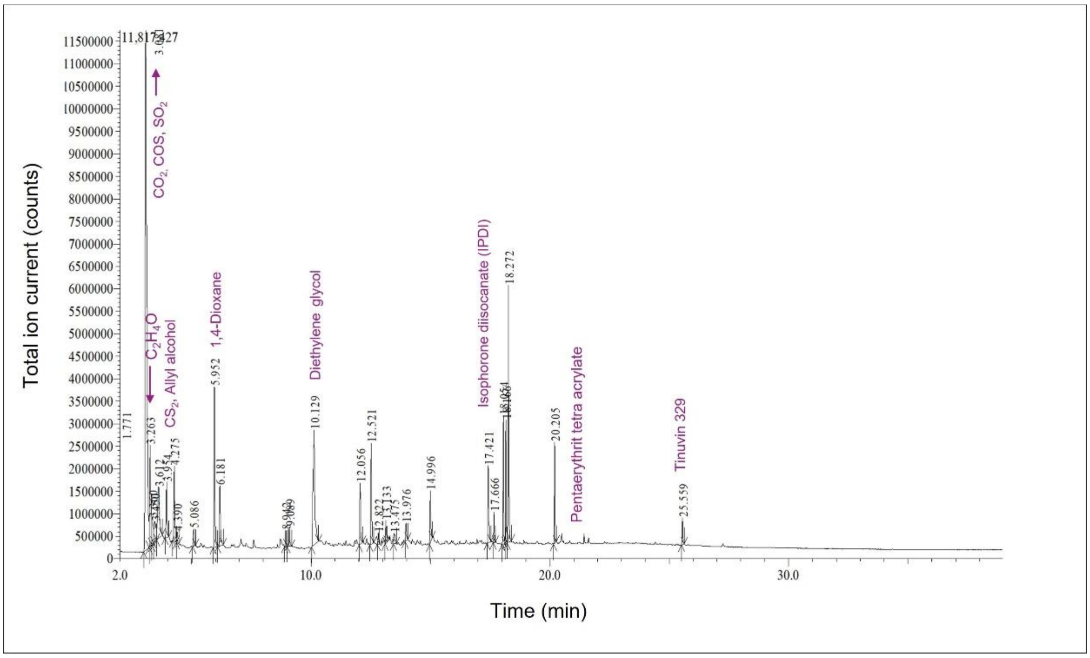

Supplementary Figure 20: Pyrolysis-gas chromatography mass spectrometry

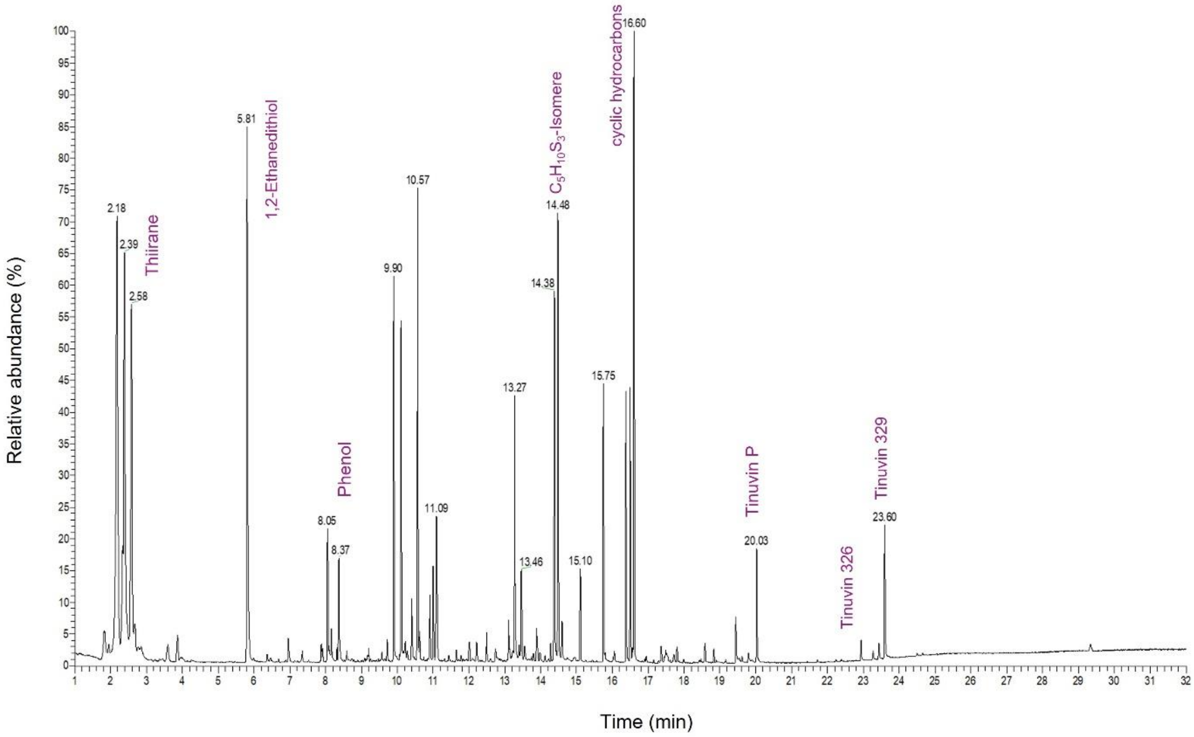

Supplementary Figure 21: Thermodesorption – gas chromatography – mass spectrometry

3. *Supplementary Tables*

Supplementary Table 1. Measured parameters Pyrolysis-GC-MS

Instrument	QP2010plus / GC2010 with Pyrolysator PY2020 (Shimadzu/Frontier Lab)
Method	Pyrolysis GC-MS
Column	30 m x 0.25 mm DB-5, Film 0.25 µm
Carrier gas	Helium
Flow rate	1.1 mL min^{-1} (constant flow)
Oven temperature	40 °C (3 min) - 12°C min^{-1} – 280 °C (22 min)
Pyrolysis temperature	550 °C
Weight in pyrolysis cup	0.5 mg
Injector temperature	250 °C
Split	1:100
Ionisation	EI (70 eV)
Mass range	A: Scan 25-700u

Supplementary Table 2. Measurement parameters thermodesorption GC-MS

Device	Trace GC Ultra / ISQ (Thermo Scientific)
Capillary column	30 m x 0.25 mm DB5, film thickness 0.25 µm
Carrier gas	Helium
Flow rate	1.1 mL min^{-1} (constant flow)
Temperature program GC	40°C (3 min) – 12 °C min^{-1} – 300 °C (16 min)
Temperature program TDU	60 °C min^{-1} – 220 °C (10 min)
Weight of samples	1 mg
Injector temperature	220 °C
Split	Split 1:30
Ionization	EI (70 eV)
Detection	Fullscan / TIC

Supplementary Table 3. Comparison table of electrochemical-driven processes for nanoplastics treatment

Electrochemical process	Principle	Removal efficiencies and materials used	(+) Advantages and (-) drawbacks	Operational cost	References
**Electrocoagulation**	Cations produced by the metal anode form micro coagulants that combine with polymer particles, from flocs and then a layer of sludge[5] or foam[6] which can be removed from the waste water.	**Nanoplastics:**<br>• 95% for PS 175.6 ± 0.83 nm [6]<br>**Microplastics:**<br>• 98% for laundry wastewater[7]<br>• 93.2% for PE (286.7 µm)[5]<br>• 91.7% for granular PMMA (6.3 µm)[5]<br>• 98.2% for fibrous CA (1–2 mm)[5]<br>• 98.4% for fibrous PP (1–2 mm)[5]<br>• 99% and more for polyester [8]<br>• 99.24% for polyethylene microbeads[9]<br>• 97.5% microplastics and heavy metals[10]	(+) easy operation<br>(+) inexpensive anode material<br>(-) does not work for small positively charged particles (90 nm)[11]<br>(-) loss of anode material, frequent replacement of the anode<br>(-) recovered particles are mixed with metal ions	• 1.32 $ m^{-3}<br>• 910 $ m^{-3} [10]	• Shen et al., 2022[5]<br>• Pawak et al., 2023[6]<br>• Akarsu and Deniz, 2020[7]<br>• Elkhatib et al., 2021[8]<br>• Perren et al., 2018[9]<br>• Xu et al., 2022[10]<br>• Tsai et al., 2023[11]
**Electrochemical adsorption**	Particles move in the electric field and adsorb at the attracting electrodes which are made of adsorbing materials[12].	• 0.707 g nano-polystyrene/g activated carbon[13]<br>• 0.322 g aged nano-polystyrene/g activated carbon[13]	(+) less chemically intensive<br>(+) easy operation<br>(-) an ion exchange membrane is needed to prevent secondary pollution [13]<br>(-) the anodes adsorption capacity is a limiting factor [13]	-	• Chen at el., 2022[12]<br>• Xiong et al., 2020[13]
**Electrokinetic separation**	Electric field driven separation through electrophoretic motion of ions and electroosmotic flow of the solvent. Dielectrophoresis is the most popular technique, in which dielectrically polarized particles move through microfluidic channels[12].	No separation yet, only redirection [14]	(+) can be used for bio(medical) waste water[12]<br>(-) no mass production possible because of complex multilayer and precision fabrication [15]<br>(-) no separation achieved yet[14]	-	• Chen et al., 2022[12]<br>• Davies et al., 2018[14]<br>• Chen et al., 2019[15]
**Electrocatalytic degradation/anodic oxidation**	Polymer particles are oxidized by strong hydroxyl radicals generated by electrolysis at the anode. An electro-peroxidation process using H_2O_2 increases the efficiency because the particles can be additionally degraded by OH which is produced at the cathode by splitting H_2O_2 and SO_4 produced from reactions with H_2O_2[12].	**Nanoplastics:**<br>•86.8% ± 1.8% for PS nanospheres (100 ± 4 nm with an EO-H_2O_2 process[16]<br>• 80.4 ± 0.93% for PS nanospheres (100 ± 4 nm)[16]<br>**Microplastics:**<br>• 89, 58 and 52% for PS (25 mm) with Na_2SO_4, NaCl and $NaNO_3$ [17]	(+) high degradation capability[12]<br>(+) high environmental compatibility[12]<br>(+) no waste or by-products for full degradation[5]<br>(-) incomplete degradation can lead to toxic or hazardous by-products[5]<br>(-) particles cannot be recycled	• 68.5, 59.9 and 57.6 $ m^{-3} for processes with Na_2SO_4, NaCl and $NaNO_3$[17]<br>• 22.1 $US m^{-3} [16]<br>• 1.6 $US m^{-3} by using a electro-peroxidation process[16]	• Chen et al., 2022[12]<br>• Kiendrebeogo et al., 2022[16]<br>• Kiendrebeogo et al., 2021[17]<br>• Shen et al., 2022[5]
**Electrophoretic deposition & Particle stabilized foam (ePhoam)**	Nanoparticles move towards the attracting electrode and produce a recoverable coating. Also, the nanoparticles attach to gas bubbles formed by water electrolysis and build particle stabilized foam that can be recovered as well.	• 98% PMMA (361 nm)<br>• 95% PMMA industrial wastewater (130 nm)<br>• 96% PS-carboxylate (295 nm)<br>• 94% PS-amidine (303 nm)<br>• 92% PS-sulfonate (137 nm)<br>• 63% PBMA-carboxylate (221 nm)<br>• 65% Eyeglass polishing wastewater (heterogeneous)<br>• 92% water-based paint (238 nm)	(+) nanoparticles stay intact and can be recycled<br>(+) proven to work for different materials<br>(+) chemical free process<br>(-) further research on continuous foam removal is needed	• 24 € m^{-3} or 27 $ m^{-3} without payback for the recovered polymers	This work

Supplementary Table 4. Comparison table of conventional, non-electrochemical-driven techniques and this present process

**Conventional process**	**Principle**	**Removal efficiencies and materials used (NPs)**	**(+) Advantages and (-) drawbacks**	**References**
**Aggregation and settling**	A chemical coagulant which neutralizes the particles charge and initiates destabilization and collision of the particles is added. The aggregated particles settle after some time and can be removed[18].	98.5% PS (50-1000 nm) with aluminium chlorohydrate[19]	(+) easy operation<br>(+) well established<br>(-) recovered particles are mixed with coagulants<br>(-) coagulation is dependent on water properties[18]	Zhang et al., 2021[19]<br>Chen et al., 2022[18]
**Aggregation and granular filtration**	A chemical coagulant which neutralizes the particles charge and initiates destabilization and collision of the particles is added[18]. Thereafter the water is passed through a granular filter medium like sand and granular activated carbon[20].	99.2% PS (124 ± 38 nm) [20]	(+) easy operation<br>(+) well established<br>(-) recovered particles are mixed with chemicals or the filter medium and cannot be recycled<br>(-) coagulation is dependent on water properties[18]	Ramirez Arenas et al., 2022[20]
**Membrane filtration**	The porous membrane allows the water and small substances like ions to pass through but prevents larger particles from passing.	89.9% PS (50 nm) with electrospun membranes[21]	(+) Charged electrospun membranes could remove PS nanoparticles from water.<br>(-) different pore sizes and charges of the membrane are required for different NPs<br>(-) conventional membrane processes are not suitable for removal of NPs[22]	Wang et al., 2020[21]<br>Mohana et al., 2021[22]
**Degradation via oxidation**	In the advanced oxidation process radicals attack the bonds of the polymer. The surface is roughed, the particle is decreased in size and increased in hydrophilicity while gradually mineralized.[18]	99.9% molecular weight degradation and 42.7% mineralization with ozonation of PS ($8*10^6$ molecular weight) in 4 h[23]	(+) no waste or by-products for full degradation[5]<br>(-) particles cannot be recycled<br>(-) incomplete degradation can lead to toxic or hazardous by-products[5]	Li et al., 2022[23]<br>Chen et al., 2022[18]<br>Shen et al., 2022[5]
**Electrophoretic deposition & Particle stabilized foam (ePhoam)**	Nanoparticles move towards the attracting electrode and produce a recoverable coating. Also, the nanoparticles attach to gas bubbles formed by water electrolysis and build particle stabilized foam that can be recovered as well.	• 98% PMMA (361 nm)<br>• 95% PMMA industrial wastewater (130 nm)<br>• 96% PS-carboxylate (295 nm)<br>• 94% PS-amidine (303 nm)<br>• 92% PS-sulfonate (137 nm)<br>• 63% PBMA-carboxylate (221 nm)<br>• 65% Eyeglass polishing wastewater (heterogeneous)<br>• 92% water-based paint (238 nm)	(+) nanoparticles stay intact and can be recycled<br>(+) proven to work for many different materials<br>(+) chemical free process<br>(-) further research on continuous foam recovery is needed	This work

Supplementary Table 5. measurement of molecular weight distributions of industrial PMMA polymers before and after the separation process using GPC technique

Sample	*M*n (g mol^{-1})	*M*w (g mol^{-1})	*Đ*
PMMA polymer before separation-dispersion	24,400	65,600	2.68
PMMA polymer after separation- recovered powder	22,700	66,100	2.91

Supplementary Table 6. Measurement parameters of evolved gas analysis characterization (EGA): a hyphenated technique of thermogravimetry and mass spectrometry (TGA-MS)

EGA	Description
TG<br>Thermogravimetry	Mass change as a function of the temperature with temporal derivative (DTG curve)
MS<br>Mass spectrometry	Emission gas analysis up to mass 50
Apparatus	STA 449 F3 Jupiter (Fa. Netzsch)<br>QMS 403 Quadro Aëolos<br>FT-IR INVENIO-S (Fa. Bruker)
Temperature interval	40 to 300 °C
Heating rate	20 K min^{-1}
Weight	≈ 64 mg
Crucible	Al_2O_3 (300 µL)
Purge gas	Helium (50 mL min^{-1})
Scales protection gas	Helium (20 mL min^{-1})

Supplementary Table 7. Measurement parameters of X-rays photo electron spectroscopy

Device	ESCALAB 250xi
Manufacturer	ThermoFisher Scientific
Source	Al k Alpha
Type	Monochromator
Energy (eV)	1486.68 eV
Voltage	15 kV
Measuring spot	650 µm (diameter)
Pass power (eV)	Standard: 200 or 20
Increment (eV)	0.1
Scans	varied, standard 10 scans

Supplementary Table 8. Results of the curve decompositions of the sum signal for the identification of the C-species and of the O-species

**Species, rough assignment**	**PMMA before separation**		**PMMA after separation**	
	**Binding energy (eV)**	**Relative amount (%)**	**Binding energy (eV)**	**Relative amount (%)**
C=O	532.6	42.2	531.6	44.4
C-OH	533.2	55.8	533.1	55.0
Residual moisture (H_2O)	535.1	2.0	534.9	0.6
C-C	284.7	55	284.6	59
C-O	286.2	27	286.1	24
O=C-O	288.6	18	288.5	17

Supplementary References

1. Lee, H. in *et al.* The Structural Effect of Electrode Mesh on Hydrogen Evolution Reaction Performance for Alkaline Water Electrolysis. *Front. Chem.* **9,** 787787; 10.3389/fchem.2021.787787 (2021).
2. European Commission, Joint Research Centre (JRC). Commission Regulation (EU) No 601/2012 of 21 June 2012 on the monitoring and reporting of greenhouse gas emissions pursuant to Directive 2003/87/EC of the European Parliament and of the CouncilText with EEA relevance.
3. El-Saftawy, A. A., Abd El Aal, S. A., Badawy, Z. M. & Soliman, B. A. Investigating wettability and optical properties of PADC polymer irradiated by low energy Ar ions. *Surf. Coat. Technol.* **253,** 249–254; 10.1016/j.surfcoat.2014.05.048 (2014).
4. Malek, M. A. & Chong, C. S. FTIR study of H2O in polyallyl diglycol carbonate. *Vib. Spectrosc.* **24,** 181–184; 10.1016/S0924-2031(00)00071-0 (2000).
5. Shen, M. *et al.* Efficient removal of microplastics from wastewater by an electrocoagulation process. *Chem. Eng. J.* **428,** 131161; 10.1016/j.cej.2021.131161 (2022).
6. Pawak, V. S., Loganathan, V. A. & Sabapathy, M. Efficient removal of nanoplastics from synthetic wastewater using electrocoagulation; *ArXiv* 10.48550/arXiv.2302.08451 (2023).
7. Akarsu, C. & Deniz, F. Electrocoagulation/Electroflotation Process for Removal of Organics and Microplastics in Laundry Wastewater. *Clean – Soil, Air, Water* **49,** 2000146; 10.1002/clen.202000146 (2021).
8. Elkhatib, D., Oyanedel-Craver, V. & Carissimi, E. Electrocoagulation applied for the removal of microplastics from wastewater treatment facilities. *Sep. Purif. Technol.* **276,** 118877; 10.1016/j.seppur.2021.118877 (2021).
9. Perren, W., Wojtasik, A. & Cai, Q. Removal of Microbeads from Wastewater Using Electrocoagulation. *ACS Omega* **3,** 3357–3364; 10.1021/acsomega.7b02037 (2018).
10. Xu, R. *et al.* Removal of microplastics and attached heavy metals from secondary effluent of wastewater treatment plant using interpenetrating bipolar plate electrocoagulation. *Sep. Purif. Technol.* **290,** 120905; 10.1016/j.seppur.2022.120905 (2022).
11. Tsai, M.-H., Chao, S.-J., Chung, K.-H., Hua, L.-C. & Huang, C. Destabilization of polystyrene nanoplastics with different surface charge and particle size by Fe electrocoagulation. *Sci. Total Env.* **872,** 162254; 10.1016/j.scitotenv.2023.162254 (2023).
12. Chen, Z., Wei, W., Liu, X. & Ni, B.-J. Emerging electrochemical techniques for identifying and removing micro/nanoplastics in urban waters. *Water Res.* **221,** 118846; 10.1016/j.watres.2022.118846 (2022).
13. Xiong, Y. *et al.* Interfacial interaction between micro/nanoplastics and typical PPCPs and nanoplastics removal via electrosorption from an aqueous solution. *Water Res.,* 116100; 10.1016/j.watres.2020.116100 (2020).
14. Davies, C. D., Yoon, E. & Crooks, R. M. Continuous Redirection and Separation of Microbeads by Faradaic Ion Concentration Polarization. *ChemElectroChem* **5,** 877–884; 10.1002/celc.201700450 (2018).
15. Chen, Q. & Yuan, Y. J. A review of polystyrene bead manipulation by dielectrophoresis. *RSC Adv.* **9,** 4963–4981; 10.1039/c8ra09017c (2019).

16. Kiendrebeogo, M., Karimi Estahbanati, M. R., Ouarda, Y., Drogui, P. & Tyagi, R. D. Electrochemical degradation of nanoplastics in water: Analysis of the role of reactive oxygen species. *Sci. Total Env.* **808,** 151897; 10.1016/j.scitotenv.2021.151897 (2022).

17. Kiendrebeogo, M., Karimi Estahbanati, M. R., Khosravanipour Mostafazadeh, A., Drogui, P. & Tyagi, R. D. Treatment of microplastics in water by anodic oxidation: A case study for polystyrene. *Environ. Pollut.* **269,** 116168; 10.1016/j.envpol.2020.116168 (2021).

18. Chen, Z., Liu, X., Wei, W., Chen, H. & Ni, B.-J. Removal of microplastics and nanoplastics from urban waters: Separation and degradation. *Water Res.* **221,** 118820; 10.1016/j.watres.2022.118820 (2022).

19. Zhang, M. *et al.* Removal of micron-scale microplastic particles from different waters with efficient tool of surface-functionalized microbubbles. *J Haz. Mater.* **404,** 124095; 10.1016/j.jhazmat.2020.124095 (2021).

20. Ramirez Arenas, L., Ramseier Gentile, S., Zimmermann, S. & Stoll, S. Fate and removal efficiency of polystyrene nanoplastics in a pilot drinking water treatment plant. *Sci. Total Env.* **813,** 152623; 10.1016/j.scitotenv.2021.152623 (2022).

21. Wang, R., Zhang, L., Chen, B. & Zhu, X. Low-pressure driven electrospun membrane with tuned surface charge for efficient removal of polystyrene nanoplastics from water. *J. Membr. Sci.* **614,** 118470; 10.1016/j.memsci.2020.118470 (2020).

22. Mohana, A. A., Farhad, S. M., Haque, N. & Pramanik, B. K. Understanding the fate of nano-plastics in wastewater treatment plants and their removal using membrane processes. *Chemosphere* **284,** 131430; 10.1016/j.chemosphere.2021.131430 (2021).

23. Li, Y. *et al.* Degradation of nano-sized polystyrene plastics by ozonation or chlorination in drinking water disinfection processes. *Chem. Eng. J.* **427,** 131690; 10.1016/j.cej.2021.131690 (2022).
