# Supplementary material for: Efficient removal of nanoplastics from industrial wastewater through synergetic electrophoretic deposition and particle-stabilized foam formation
Source: Nat Commun. 2024 Jun 27;15:5437. doi: 10.1038/s41467-024-48142-2 (PMC11211448; doi:10.1038/s41467-024-48142-2)
Supplement: Supplementary file 3 — Description of Additional Supplementary Files [file 41467_2024_48142_MOESM3_ESM.pdf]

Description of Additional Supplementary Files

File Name: Supplementary Movie 1
Description: Formation of particle-stabilized foam in the batch cell setup.

File Name: Supplementary Movie 2
Description: The anode surface after removal from the cell using the electroflotation setup.

File Name: Supplementary Movie 3
Description: Size and formation rate of the oxygen bubbles on the stainless steel anode using a high-speed camera.

File Name: Supplementary Movie 4
Description: Size and formation rate of the oxygen bubbles on the flat DSA anode using a high-speed camera.

File Name: Supplementary Movie 5
Description: Size and formation rate of the oxygen bubbles on the mesh DSA anode using a high-speed camera.

File Name: Supplementary Movie 6
Description: Removal of particles in the continuous roller setup, while the electrode surface is re-immersed and replenished continuously.
